# Incidence of Concomitant Semicircular Canal Dehiscence With Otosclerosis

**DOI:** 10.1097/ONO.0000000000000012

**Published:** 2022-06-24

**Authors:** Brian A. Walker, Ryan M. Thorwarth, Lindsey L. Stull, Joseph M. Hoxworth, Nicholas L. Deep, Peter A. Weisskopf

**Affiliations:** 1 Department of Otorhinolaryngology, The Mayo Clinic in Arizona, Phoenix, AZ; 2 Department of Radiology, Division of Neuroradiology, The Mayo Clinic in Arizona, Phoenix, AZ; 3 Department of Otorhinolaryngology, Division of Neurotology, The Mayo Clinic in Arizona, Phoenix, AZ.

**Keywords:** CT temporal bone, Otosclerosis, Semicircular canal dehiscence

## Abstract

**Objective:**

The concurrence of otosclerosis and superior semicircular canal dehiscence (SSCD) presents a diagnostic challenge and failure to differentiate between these 2 diagnoses results in mischaracterization and unsuccessful surgery. The objective of this study is to identify the incidence of SSCD in patients who have computed tomography (CT) evidence of otosclerosis.

**Study Design:**

Retrospective chart review.

**Setting:**

Tertiary referral hospital.

**Patients:**

Adults with CT scan of the temporal bone diagnosed with radiological unilateral or bilateral fenestral otosclerosis from January 1995 to April 2018.

**Methods:**

Retrospective review of patient imaging from a multi-center tertiary-referral health system from January 1995 to April 2018. Imaging was reviewed to quantify the incidence of SSCD among patients with CT-diagnosed bilateral fenestral otosclerosis. Poor quality imaging was excluded from review.

**Results:**

One-thousand two-hundred eight patients (1214 CT scans) were identified with otosclerosis, of which 373 were diagnosed with fenestral otosclerosis (663 ears) with imaging of sufficient quality for review. This population was predominantly female (57.2%) with bilateral fenestral otosclerosis (78%). Of these, 23 ears (3.5%) had definitive evidence of SSCD, with an additional 15 ears (2.3%) with possible radiographic evidence of SSCD. There was no significant difference in laterality between the SSCD and otosclerosis.

**Conclusions:**

Among 373 patients with fenestral otosclerosis per CT temporal bone imaging at a tertiary referral hospital, as many as 8.3% of patients had radiographic evidence of SSCD. Given this incidence, it continues to be important to consider SSCD when diagnosing and treating otosclerosis.

The concurrence of otosclerosis and superior semicircular canal dehiscence (SSCD) presents a diagnostic challenge. Both are a result of an alteration in the number of functioning “windows” into the inner ear, and as such can have overlapping symptomology, which complicates diagnosis and treatment. There has been increasing interest in the concurrence of SSCD and otosclerosis over recent years (1,2). With respect to diagnosis, stapes footplate fixation at the oval window relegates the 3-window status back to a 2 window status and may reduce or abolish the third window phenomena. This may mask symptoms of SSCD, making diagnosis of clinical SSCD syndrome difficult in the presence of otosclerosis (1,3–5). In addition to this diagnostic challenge, there are 2 major consequences of treatment:

1. Correcting stapes fixation, but not the third window, may result in failure to close the air-bone gap (6).

2. Correcting stapes fixation in the setting of SSCD may “unmask” the third window phenomenon and result in SSCD syndrome (7,8).

Therefore, in patients with concurrent disease, there is a possibility of failing to recognize both diagnoses with subsequent treatment failure and symptomatic worsening.

In addition to patients that truly have both disease processes, some argue that SSCD alone, in the absence of otosclerosis, can present with predominantly auditory complaints and mimic otosclerosis in presentation. This may also lead to misdiagnosis and middle ear surgery that is ultimately ineffective (9–12).

There are many relevant examples in the literature that demonstrate these issues. Most common is the report of failure to resolve conductive hearing loss upon stapes surgery in patients with SSCD and otosclerosis (6). There are also multiple descriptions of patients who failed multiple stapes procedures that subsequently were found to have SSCD (2,5,12–14). For these reports, it is often unclear if the patient was misdiagnosed with otosclerosis originally or if the persistent third window was the cause for failure to close the air-bone gap.

Given these reports, it is essential to accurately identify patients with both otosclerosis and SSCD in order to guide clinical decision-making prior to any surgical intervention. Acoustic reflexes, vestibular evoked myogenic potentials testing, and a detailed history and physical have aided in distinguishing these diagnoses; however, the gold standard for characterization of both SSCD and otosclerosis is high-resolution computed tomography (CT) (5,15). This is not always performed, since otosclerosis remains a clinical diagnosis. In otosclerosis, CT is largely only used when there is concern for anatomical abnormality, which may alter the risk/benefit profile of surgery and guide the surgical plan (16–23). As such, it is important to identify what proportion of patients with otosclerosis may also have SSCD in order to guide the need for imaging preoperatively.

Concurrence of otosclerosis and SSCD has been estimated at 150 in 100,000 people in the general population based on the prevalence of each individual disease process (2). It has been shown that in those patients with a tentative diagnosis of otosclerosis based on clinical exam and audiometry, 5.3% had SSCD on CT (9). What has not been described is the radiographic concurrence of both disease processes, irrespective of the clinical diagnosis. Herein, we evaluate the incidence of SSCD within a large series of CT studies with evidence of fenestral otosclerosis.

## MATERIALS AND METHODS

**FIG. 1.**
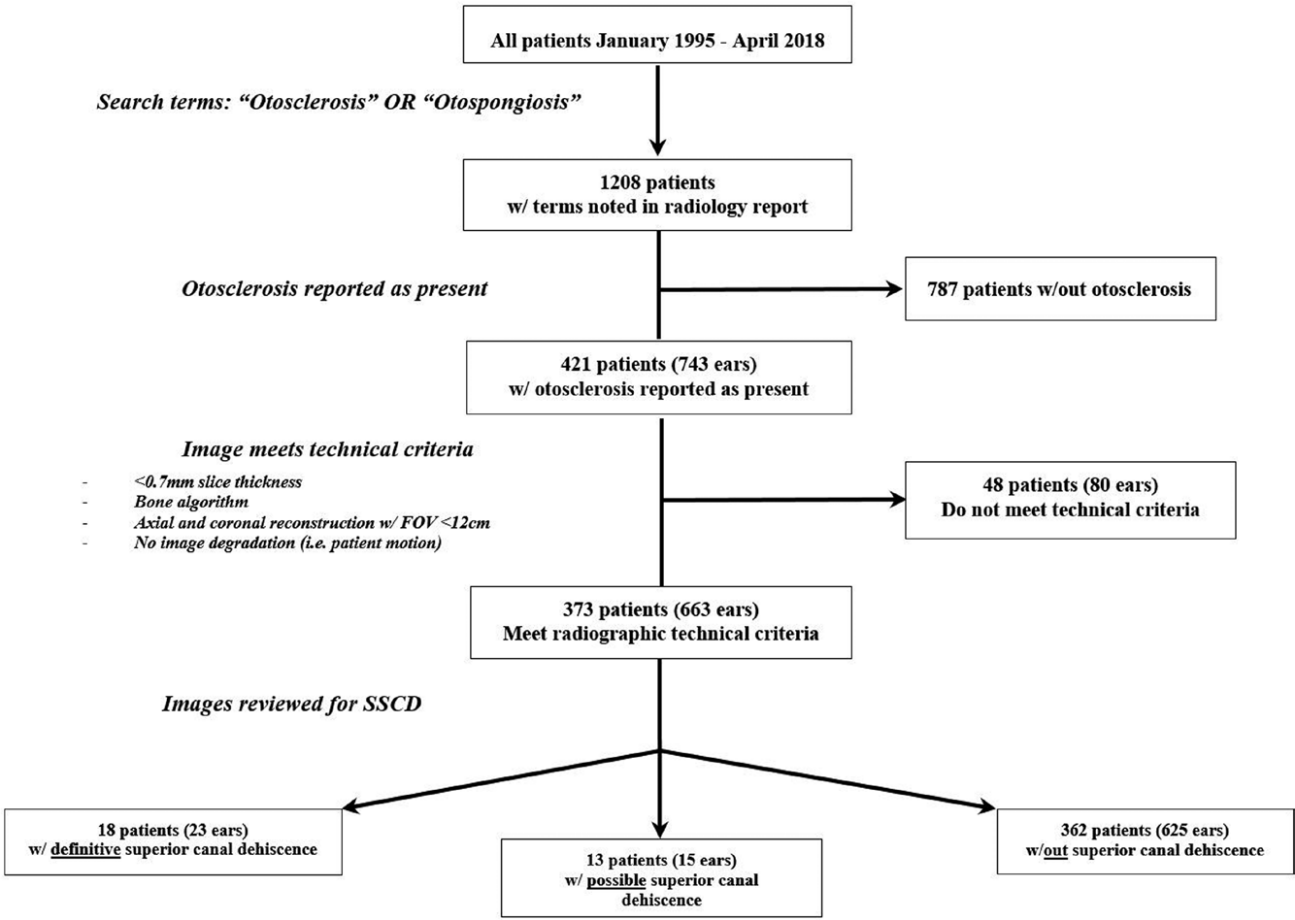
Flow diagram for chart review and image review process. FOV indicates field of view; SSCD, superior semicircular canal dehiscence.

This study was deemed institutional review board (IRB) exempt after preliminary review by the IRB. Patient records from a multi-state tertiary referral institution were queried for radiology reports containing the term “otosclerosis” and “otospongiosis” between January 1, 1995, and April 1, 2018. This initial search did not specify fenestral versus non-fenestral location or imaging type. All temporal bone CT scans were then reviewed to include only those cases with imaging evidence of fenestral otosclerosis and to document whether this was unilateral or bilateral (Fig. 1).

In order to make certain that SSCD could be confidently assessed, the CT scans for the subjects with confirmed fenestral otosclerosis were reviewed for technical adequacy and image quality. Inclusion criteria dictated that the image was a dedicated temporal bone CT with helical acquisition on a multidetector CT scanner with a slice thickness <0.7 mm using a bone algorithm. Axial and coronal reconstructions of each temporal bone side had to have a field of view ≤12 cm. Additionally, image quality had to be deemed adequate by the neuroradiologist with no degradation from beam-hardening streak artifact, patient motion, or other concerning features. The availability of Poschl/Stenver oblique reconstructions was noted but absence did not exclude the patient since these are not necessary for diagnosis of SSCD in the vast majority of cases (24).

All images that met technical inclusion criteria were reviewed by 1 neuroradiologist who made an initial assessment of the thickness of the intervening bone between the superior canal and the middle cranial fossa. If this was ≥0.6 mm, then SSCD was excluded, and no further review was necessary. The remaining cases with thickness <0.6 mm underwent joint review by both the neuroradiologist and a neurotologist and were categorized as having no superior canal dehiscence, clear dehiscence, or possible/equivocal dehiscence based upon their consensus opinion.

## RESULTS

Otosclerosis or otospongiosis was identified in the radiology reports of 1208 patients. Of these 1208 patients, 1214 radiology reports required review, as some patients had more than 1 study completed. Review of these records yielded 421 patients whose diagnostic report was positive for fenestral otosclerosis on one or both sides. Of the 421 patients with radiographic fenestral otosclerosis, 373 patients (663 ears) met minimum technical inclusion criteria. Of note, 54 of those patients (96 ears) met technical inclusion criteria but did not have oblique reconstructions available.

Of these 373 patients (663 ears), the mean age at the time of imaging was 54 years of age but ranged from 7 to 94 years old. A female predominance (57.2%) was also noted within this population (Table 1). The vast majority, 290 patients (78%; 580 ears) had bilateral otosclerosis present (Table 2).

**TABLE 1.** Demographic data for patients with otosclerosis and SSCD

Variable	Otosclerosis (N = 373)	Otosclerosis + definitive SSCD (N = 18)
Age		
Mean (SD)	54 (15.3)	56 (13)
Range	7–94	35–82
Gender		
Female	214 (57.2%)	12 (66%)
Male	159 (42.8%)	6 (33%)

SSCD indicates superior semicircular canal dehiscence.

**TABLE 2.** Descriptive statistics for incidence of otosclerosis and SSCD

Laterality of Otosclerosis	No. patients (N = 373)	No. ears (N = 663)
Left side only	37 (10%)	37 (5.6%)
Right side only	46 (12%)	46 (6.9%)
Bilateral	290 (78%)	580 (87.5%)
**Concurrent Superior Canal Dehiscence**	**No. patients (N = 373)**	**No. ears (N = 663)**
Possible dehiscence by consensus	18 (4.8%)	15 (2.3%)^*a*^
Definite dehiscence by consensus	13 (3.5%)	23 (3.5%)^*b*^
No superior canal dehiscence	342 (91.7%)	625 (94.3%)

^*a*^Two patients did not have oblique reconstructions available.

^*b*^One patient did not have oblique reconstructions available.

SSCD indicates superior semicircular canal dehiscence.

Of the 663 ears with radiographic otosclerosis, 23 ears (3.5%) had definitive radiographic evidence of SSCD. An additional 15 ears (2.3%) had possible radiographic dehiscence. Only 2 of the 13 patients with possible dehiscence did not have oblique reconstructions available for review. Incidence of SSCD based on laterality of otosclerosis was similar between patients right (4%) and bilateral (5%) otosclerosis. No cases of left otosclerosis had concurrent SSCD. Among patients with bilateral otosclerosis, SSCD occurred with equal frequency on the left (1.3%), right (1.6%), and bilateral (1.3%) ears (Table 3).

**TABLE 3.** Laterality for concurrent otosclerosis and SSCD

Laterality of Otosclerosis	Laterality of definitive SSCD		
	Left side only	Right side only	Bilateral
Left side only (n = 37)	0 (0%)	N/A	N/A
Right side only (n = 46)	N/A	2 (0.5%)	N/A
Bilateral (n =290)	5 (1.3%)	6 (1.6%)	5 (1.3%)

SSCD indicates superior semicircular canal dehiscence.

## DISCUSSION

The precise incidence of otosclerosis with concurrent SSCD is unknown (1,2,25,26). Herein, we evaluated the incidence of SSCD in 373 patients (663 ears) with radiologic evidence of otosclerosis. This yielded an incidence of SSCD at 4.8% to 8.3% of patients (3.5%–5.8% of ears) with CT-diagnosed otosclerosis.

Prior attempts at characterizing the incidence of SSCD in the general population have been performed. These reports include histological and radiological studies of patients with and without subjective criteria of SSCD. As a result, the incidence of SSCD is widely variable, ranging from 0.5% to 9% of patients, depending on the methods used (27–32). An important caveat to consider is that incidences varied based on the type of study performed, with radiological studies identifying an increased rate of SSCD in comparison to histological studies (2,33–36). This has been proposed to be due to a bias towards overdiagnosis when using CT scans (27,29,31). Our findings regarding the incidence of SSCD in otosclerosis are comparable to similar studies of SSCD incidence in CT temporal bones with a rate of 4.8%–8.3% of patients with potential SSCD. The variability in our own reported incidence is a result of a large quantity of imaging, which was deemed “possible radiographic dehiscence,” thus illustrating the effect overdiagnosis could have on the incidence of the condition in a population of CT surveys.

Concurrence of otosclerosis and SSCD has been estimated as 150 in 100,000 (0.0015%) people in the general population based on the prevalence of each individual disease process (2). Given that our study population included only individuals who were diagnosed with otosclerosis on imaging, we are unable to further remark on the incidence within the asymptomatic general population. However, as discussed previously, the supposed incidence of SSCD in the general population ranges from 0.5% to 9% (27–32). Considering SSCD development independent of otosclerosis development and assuming that our population is representative of the general population, we would anticipate that 2 to 33 of the patients within our cohort would also have SSCD. We report 18 patients (4.8%) with definitive SSCD and 13 additional patients with possible SSCD within our cohort, resulting in 31 possible cases of SSCD. This number of patients matches what would be expected from a sample of the general population and potentially alludes to the lack of an association between these diagnoses.

Alternatively, another study of imaging in patients who were clinically diagnosed with otosclerosis identified an incidence of SSCD in 5.3% of patients (9). Our observations agree and build upon this observation. Given that our data suggest an incidence between 4.8% and 8.3% of patients, it is possible that the true incidence lies somewhere within this range.

Ultimately, our study is limited due to its retrospective nature and small population size, which limits subgroup analysis. Patients undergoing CT temporal bone scans for any indication may not represent the general population or those suspected of otosclerosis/superior canal dehiscence clinically. There were also certain limitations from a radiological perspective in accruing subjects over such a lengthy study duration, but we attempted to mitigate this through the application of strict technical inclusion and exclusion criteria. Given the nature of our study, a significant minority of scans did not have Stenver and Poschl reformats. However, these have been found to only impact the radiologic diagnosis in equivocal or confusing cases, so it is noteworthy that only 2 of 13 subjects with “possible radiographic dehiscence” lacked these oblique reconstructions (24). Another important limitation of our study is that both otosclerosis and SSCD syndrome can occur radiologically in patients without clinical symptoms. Consideration was given to studying only clinically symptomatic otosclerosis patients; however, it is not our practice to routinely image these patients with CT temporal bone scans. Instead, we identified patients with radiographic evidence of fenestral otosclerosis, regardless of their primary diagnosis, clinical symptoms, or indication for imaging. Therefore, we cannot clarify whether any of the identified CT findings were clinically significant.

The strength of our analysis is in isolating patients with radiographic otosclerosis findings and a large sample cohort while avoiding other confounding variables and using this to further characterize the relationship between the diagnosis of SSCD within patients with CT-identifiable otosclerosis. This provides needed insight into how much weight the diagnosis of SSCD should be given when considering patients presenting with otosclerosis or unclear symptoms. With this information, we believe that surgeons can more carefully consider the use of CT imaging prior to surgical intervention and utilize that modality for patients with additional suspicious symptoms or history.

## CONCLUSIONS

Otosclerosis and SSCD are distinct yet related otologic pathologies, which, when concurrent, present unique challenges in diagnosis and treatment. Failure to recognize concurrence of these diagnoses in a patient leads to surgical failure, worsening symptoms, and patient dissatisfaction. Within a population of patients with CT evidence of fenestral otosclerosis, we identified an incidence of SSCD of 4.8% to 8.3% of patients. Given this information, surgeons should carefully evaluate for SSCD in patients with otosclerosis to appropriately plan and carry out the procedure that will benefit the patient.

## FUNDING SOURCES

None declared.

## CONFLICT OF INTEREST

None declared.

## DATA AVAILABILITY STATEMENT

The data from this study is available for review. Please send all requests to the corresponding author.
